# LGR5 Activates Noncanonical Wnt Signaling and Inhibits Aldosterone Production in the Human Adrenal

**DOI:** 10.1210/jc.2015-1734

**Published:** 2015-04-27

**Authors:** Lalarukh Haris Shaikh, Junhua Zhou, Ada E. D. Teo, Sumedha Garg, Sudeshna Guha Neogi, Nichola Figg, Giles S. Yeo, Haixiang Yu, Janet J. Maguire, Wanfeng Zhao, Martin R. Bennett, Elena A. B. Azizan, Anthony P. Davenport, Grahame McKenzie, Morris J. Brown

**Affiliations:** Clinical Pharmacology Unit (L.H.S., J.Z., A.E.D.T., S.G., J.J.M., E.A.B.A., A.P.D., M.J.B.) and Cardiovascular Division (N.F., H.Y., M.R.B.), Department of Medicine, University of Cambridge, Cambridge National Institute for Health Research (S.G.N.), Biomedical Research Centre, Department of Clinical Biochemistry, Addenbrooke's Hospital, University of Cambridge Metabolic Research Laboratories (G.S.Y.), Institute of Metabolic Science, Addenbrooke's Hospital, and Human Research Tissue Bank (W.Z.), Cambridge University Hospitals Foundation Trust, Addenbrooke's Hospital, Cambridge CB2 0QQ, United Kingdom; Department of Medicine (E.A.B.A.), Faculty of Medicine, The National University of Malaysia Medical Centre, Kuala Lumpur 56000, Malaysia; and Medical Research Council Cancer Unit (G.M.), University of Cambridge, Cambridge CB2 0XZ, United Kingdom

## Abstract

**Context:**

Aldosterone synthesis and cellularity in the human adrenal zona glomerulosa (ZG) is sparse and patchy, presumably due to salt excess. The frequency of somatic mutations causing aldosterone-producing adenomas (APAs) may be a consequence of protection from cell loss by constitutive aldosterone production.

**Objective:**

The objective of the study was to delineate a process in human ZG, which may regulate both aldosterone production and cell turnover.

**Design:**

This study included a comparison of 20 pairs of ZG and zona fasciculata transcriptomes from adrenals adjacent to an APA (n = 13) or a pheochromocytoma (n = 7).

**Interventions:**

Interventions included an overexpression of the top ZG gene (*LGR5*) or stimulation by its ligand (R-spondin-3).

**Main Outcome Measures:**

A transcriptome profile of ZG and zona fasciculata and aldosterone production, cell kinetic measurements, and Wnt signaling activity of *LGR5* transfected or R-spondin-3-stimulated cells were measured.

**Results:**

*LGR5* was the top gene up-regulated in ZG (25-fold). The gene for its cognate ligand R-spondin-3, *RSPO3*, was 5-fold up-regulated. In total, 18 genes associated with the Wnt pathway were greater than 2-fold up-regulated. ZG selectivity of *LGR5*, and its absence in most APAs, were confirmed by quantitative PCR and immunohistochemistry. Both R-spondin-3 stimulation and *LGR5* transfection of human adrenal cells suppressed aldosterone production. There was reduced proliferation and increased apoptosis of transfected cells, and the noncanonical activator protein-1/Jun pathway was stimulated more than the canonical Wnt pathway (3-fold vs 1.3-fold). ZG of adrenal sections stained positive for apoptosis markers.

**Conclusion:**

*LGR5* is the most selectively expressed gene in human ZG and reduces aldosterone production and cell number. Such conditions may favor cells whose somatic mutation reverses aldosterone inhibition and cell loss.

Since their discovery by Conn nearly 60 years ago, aldosterone-producing adenomas (APAs) have been regarded as infrequent: less than 1% of all hypertension; recent estimates of prevalence, however, have risen to several times this figure (1). Most APAs, though, are diagnosed too late for complete cure of hypertension (2), and there is a need for better-tolerated drugs that block the increased aldosterone production. Current treatments either compete with aldosterone for its receptor or inhibit the Na^+^ channel downstream. In addition to the limitations of efficacy or selectivity, such drugs increase aldosterone production (3). Novel drug targets can be identified by the discovery of either a gene whose gain-of-function mutation increases aldosterone production in APAs or pathways coupled to inhibition of aldosterone in the normal adrenal.

APAs are often a heterogeneous mixture of cells; paradoxically, the cells of classical Conn's tumors appear more like cortisol-producing zona fasciculata (ZF) cells than the supposedly aldosterone-producing cells of normal zona glomerulosa (ZG) (4, 5). This paradox may be resolved by the frequent finding of small APAs (often missed on conventional adrenal imaging) that consist mainly of compact cells and express genes, such as *NPNT* (encoding for nephronectin), not expressed in the ZF of human adrenals (6, 7). Exome sequencing of ten such ZG-like APAs led us to identify somatic mutations in three genes, encoding caveolin-1.3, Na^+^, K^+^-ATPase, and β-catenin (7); no ZG-like APAs have yet been found to have one of the *KCNJ5* mutations common in larger, more ZF-like APAs (8–11). The small size of many of these ZG-like APAs, sometimes documented as unchanged in serial computed tomography scans over many years, inferred limited growth before diagnosis. This observation suggested to us that cells in a ZG-like APA may derive a survival advantage from constitutive aldosterone production, rather than cell division, thus explaining the frequency of somatic mutations, 19 different mutations in caveolin-1.3 alone, which switch on constitutive aldosterone production (7, 11, 12).

In most species, ZG is unusually proliferative for an endocrine tissue, stimulated by the renin-angiotensin II response to salt depletion; but in typically salt-loaded humans, the reverse appears true (13, 14). We hypothesized the existence of a local process, in addition to withdrawal of renin, which might suppress ZG cell steroidogenesis or proliferation when not required. Previous comparisons of human adrenal ZG and ZF have depended on techniques, such as immunohistochemistry (IHC) and in situ hybridization that require investigation of specific candidate molecules (15, 16). The striking difference in appearance, after cresyl violet staining, between lipid-poor and lipid-rich cells of ZG and ZF (6), enabled us to carry out laser capture microdissection of snap-frozen fresh adrenal sections and thus whole-transcript expression analysis and profiling of ZG and ZF. The resulting top ZG gene identified, *LGR5*, is postulated to be key to this local suppression process of ZG cell activity, highlighting both a possible novel drug target that can inhibit aldosterone production and a possible link between the frequency of APAs and aldosterone secretion.

## Materials and Methods

### Study design

To determine genes and pathways regulating aldosterone production, transcriptome profiles of supposedly aldosterone-producing ZG was compared with its paired cortisol-producing ZF from 20 human adrenals. The detail method of the microarray assay and clinical demographics of the patients involved is as described previously (17) and is briefly described in Supplemental Methods. Results were represented in heatmaps with hierarchal clustering of genes and array performed by Cluster 3.0 (18) and visualization by Java TreeView (19). Adrenals were collected within 30 minutes of adrenalectomy (due to an APA or pheochromocytoma) with local ethical approval and informed consent from the patients. Adrenals immediately underwent collagenase dispersion for primary cell culture or were stored at −70°C for RNA analyses, histology and IHC.

Comparison of ZG and ZF transcriptome profile led us to hypothesize an important role for *LGR5* (the top ZG gene identified) and the Wnt signaling pathway in the regulation of aldosterone production in the human adrenal. The hypothesis was tested in the human adrenocortical carcinoma cell line, H295R, and in primary human adrenal cell cultures. The primary outputs of cell culture experiments were aldosterone secretion into the supernatant of cultures and quantitative PCR (qPCR) for *CYP11B2* expression (encoding for the enzyme aldosterone synthase). These were measured in response to key proteins modulating the Wnt signaling pathway, administered either externally or by transfection of cDNA constructs.

### Laser capture microdissection (LCM)

LCM was used to obtain samples of ZF and ZG cells from adrenal tissue adjacent to APAs or pheochromocytomas as previously described (5). For differentiation of ZG from ZF, sections were stained with cresyl violet using the LCM staining kit (AM1935; Ambion).

### Quantification of mRNA expression

Cells were kept in RNAlater and TRIzol (both from Ambion) until extracted for RNA. Total DNA-free RNA was isolated using the PureLink RNA minikit with the PureLink deoxyribonuclease set (Life Technologies) according to manufacturer's instructions. Reverse transcription was performed using the reverse transcriptase system (Promega) with a 1:1 mixture of random hexamer and oligodeoxythymidine primers according to the manufacturer's instructions. mRNA expression of genes of interest was quantified using TaqMan probes (Applied Biosystems), and *CYP11B2* expression was quantified using custom-made TaqMan probes that had been validated for specificity (20). The housekeeping 18S rRNA (Applied Biosystems) was used for normalization.

### Immunohistochemistry

IHC was performed on formalin-fixed, paraffin-embedded human adrenal sections (4 μm) using a chromogen-based detection system, 3,3′-diaminobenzidine. Commercial antibodies to LGR5 (number TA503316; Origene; 1:300 dilution), β-catenin (number 610154; BD Transduction Laboratories; 1:300 dilution), lymphoid enhancer binding factor (LEF)-1 (number ab53293; Abcam plc; 1:100), c-JUN (number ab32137; Abcam; 1:250 dilution), and phospho-c-JUN (number SC-822; Santa Cruz Biotechnology; 1:100) were used on slides selected from patient adrenals included in the microarray. Negative controls, in which primary and then secondary antibodies were omitted, were used and resulted in the absence of staining. Slides were counterstained with hematoxylin solution (Harris modified; Sigma-Aldrich). To negate cross-reactivity of LGR5 antibody with LGR5's close homologs, LGR4 and LGR6, additional anti-LGR5 antibodies (Novus Biologicals; NBP128904; Abcam; ab75732) that binds to epitopes that through basic local alignment search tool analysis (National Center for Biotechnology Information, Bethesda, Maryland) aligned specifically to *LGR5* but differs in the sequence to *LGR4* and *LGR6* were also tested in serial human adrenal sections.

Apoptosis was examined using terminal deoxynucleotidyl transferase-mediated deoxyuridine triphosphate nick end labeling (TUNEL) staining visualized by a rhodamine-labeled antidigoxigenin antibody (number 11093088910; Roche Diagnostics Corp), and proliferation was captured using an anti-Ki67 antigen (number M7240; Dako; 1:400 dilution) according to the manufacturer's instructions. Images were captured using a standard bright-field microscope, a U-TV1-X digital camera and CellD software (Olympus).

### Cell culture experiments

H295R human adrenocortical carcinoma cells and primary adrenocortical cells were cultured in DMEM/Nutrient F-12 Ham supplemented with 10% fetal calf serum, 100 U penicillin, 0.1 mg/mL streptomycin, 0.4 mM L-glutamine, and insulin-transferrin-sodium selenite medium at 37°C in 5% CO_2_ as previously described (17). Cells were transfected with vector controls, pCMV6-AC-GFP (Origene) or pcDNA3.1/His A (Invitrogen), and green fluorescent protein (GFP)-tagged *LGR5* (Origene; 600 ng/μL). Transfections were carried out using Cell Line Nucleofector Kit R (VACA-1001; Lonza) according to the manufacturer's guidelines. Cell fate assays were performed in *LGR5*-transfected cells as described in Supplemental Methods. Cells were silenced using either ON-TARGETplus non-targeting small interfering RNA (siRNA) as control or SMART pool: ON-TARGET plus *LGR5* siRNA (Dharmacon; 50 nM). Transfected cells were plated into 24-well plates (100 000 cells/well) in 0.25 mL of growth medium. After 24 hours of transfection, H295R cells were serum deprived in unsupplemented medium for 24 hours, and further drug treatments were carried out with 8 hours incubation from this point. Recombinant hR-spondin-3 (Sino Biological Inc and R&D Systems) was used where specified at 100 nM. Supernatants for aldosterone concentration measurement were collected after respective 8-hour treatments, and cells were harvested for analysis of mRNA. Aldosterone concentrations were determined by a commercially available ^125^I RIA (Diagnostic Products Corp) as detailed in Supplemental Methods.

### Wnt signaling pathway activity assays

T cell transcription factor (TCF)/LEF response was measured using Cignal TCF/LEF reporter (luc) kit (SABiosciences). Activator protein 1 (AP1)/JUN pathway response was measured using a construct created by cloning an oligo containing seven copies of the AP1 binding element (TGACTAA) into a luciferase construct pGL4.10 (luc2) (Promega) at its *Kpn*I and *Xho*I sites. pRL-TK was used for normalization (Promega). nuclear factor of activated T cells (NFAT) response was also measured using a luciferase reporter construct (plasmid number 10959; Addgene).

Plasmids modulating Wnt signaling pathway were used as comparator to show effect on aldosterone of activation or suppression of the canonical pathway. To activate the pathway, pcDNA3 δN47 β-catenin was used; and to suppress the pathway, pcDNA/Myc δN TCF4 (both from Addgene) were used. Where indicated, the vector control, pcDNA3.1-V5His (Invitrogen), was used. To measure luciferase (and renilla for normalization), a Dual-Glo luciferase assay system (Promega) was used according to the manufacturer's protocol. The selective porcine inhibitor, LGK-974 (1 μM; Selleck Chemicals) (21), and the selective c-Jun N-terminal kinase (JNK) inhibitor, JNK-IN-8 (1 μM, Selleck Chemicals) (22), were used to analyze the effect of blocking, respectively, all Wnt secretion or just the noncanonical Wnt signaling pathway.

### Statistical analysis

Unless otherwise stated, results are expressed as mean values ± SEM. Statistical analysis was performed using the Student's unpaired *t* test or ANOVA on standard statistical software.

## Results

### *LGR5* and several other genes associated with the Wnt signaling pathway are selectively and abundantly expressed in human adrenal ZG

**Figure 1.**
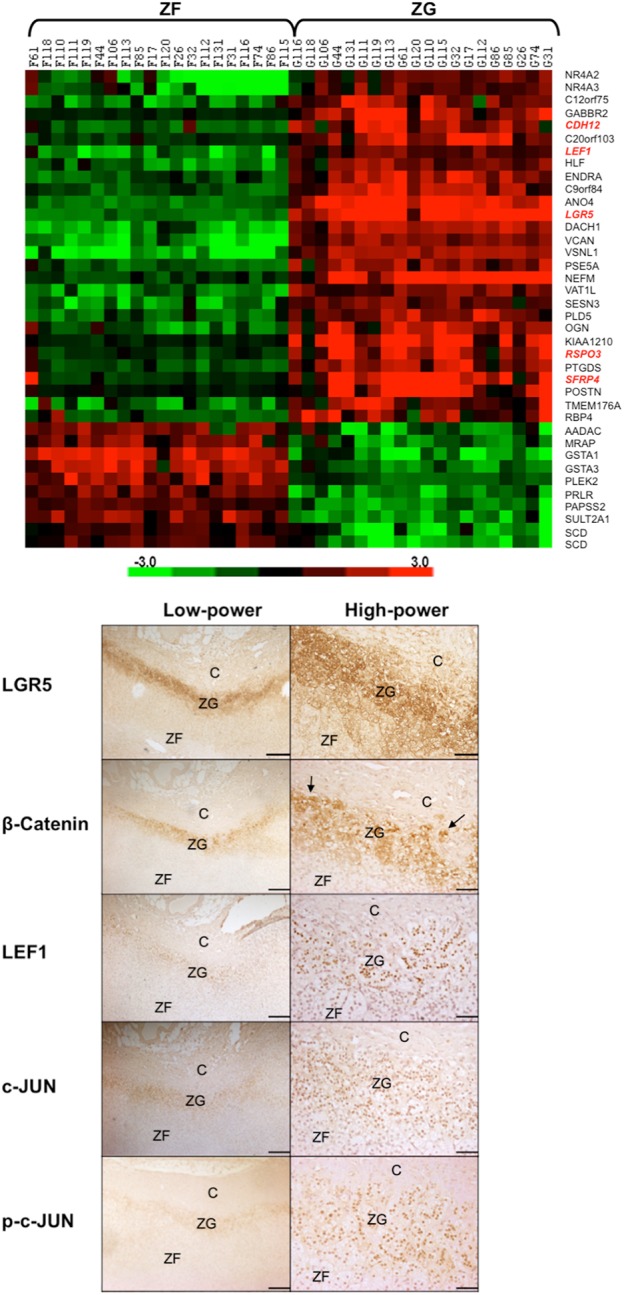
Wnt-related genes show selective expression in the ZG of human adrenal. A, Heat map of 37 genes (shown on the vertical axis), which are 5-fold or greater differentially expressed in ZG and ZF. Genes previously associated with the Wnt signaling pathway are highlighted in red. On the horizontal axis, each pair of ZG and ZF from the 20 adrenals are anonymized and numbered. Probe set and detailed microarray information are available from the National Center for Biotechnology Information Gene Expression Omnibus under accession number GSE64957. B, IHC localization of LGR5 and downstream Wnt signaling proteins in the ZG: from the canonical β-catenin pathway, β-catenin and LEF1, and from the noncanonical AP1/JUN pathway, c-JUN and p-c-JUN. IHC was performed on formalin-fixed, paraffin-embedded human adrenal sections (4 μm) using a chromogen-based detection system (3,3'-Diaminobenzidine), which results in a positive brown staining. Interestingly, β-catenin staining was mostly membranous or cytoplasmic (rather than nuclear staining as highlighted by the arrows), indicating limited canonical Wnt activation. Pictures are representative of six normal adrenal sections that were used in our microarray study; four from primary hyperaldosteronism patients and two from pheochromocytoma patients. Scale bar, 500 μm (low power) and 100 μm (high power). C, capsule.

Transcriptome analysis by Affymetrix microarray identified 213 genes, which were between 2- and 25-fold more abundant in human adrenal ZG than their paired ZF (Supplemental Figure 1). The top hit was *LGR5* (*P* = 1.6 × 10^−23^; Figure 1A and Table 1) (17), which in several tissues is recognized as a stem-cell marker (23, 24). The possibility of *LGR5* playing a key role in ZG was supported by the finding that the expression of the gene for its cognate ligand R-spondin-3, *RSPO3*, was also up-regulated in ZG by 5-fold (*P* = 3 × 10^−11^). Overall, 18 of the 213 ZG genes encode previously identified proteins associated with the Wnt signaling pathway (Table 1). By contrast, only 1 of the 117 ZF genes (those > 2-fold more abundant in ZF than ZG) encodes for a known protein from this Wnt signaling pathway.

**Table 1.** Wnt Genes With Greater Than 2-Fold Up-Regulation in ZG Compared With ZF, Ranked in Order of Fold Change (ZG vs ZF)

Rank	Gene Name*	Symbol	*P* Value	Fold Change
1	Leucine-rich repeat-containing G protein-coupled receptor 5	*LGR5*	1.6 × 10^−23^	25.0
9	Secreted frizzled-related protein 4	*SFRP4*	2.3 × 10^−10^	8.7
17	Lymphoid enhancer-binding factor 1	*LEF1*	4.3 × 10^−15^	5.9
19	Cadherin 12, type2 (N-cadherin 2)	*CDH12*	4.4 × 10^−10^	5.8
27	R-spondin 3 homolog (*Xenopus laevis*)	*RSPO3*	3.3 × 10^−11^	5.3
44	FBJ murine osteosarcoma viral oncogene homolog B	*FOSB*	5.7 × 10^−6^	3.8
53	Frizzled-related protein	*FRZB*	2.4 × 10^−8^	3.5
76	Glypican 3	*GPC3*	1.7 × 10^−6^	3.1
89	Transcription factor 21	*TCF21*	2.4 × 10^−5^	2.9
90	Jun B protooncogene	*JUNB*	5.6 × 10^−6^	2.9
92	Claudin 1	*CLDN1*	2.3 × 10^−10^	2.8
98	FBJ murine osteosarcoma viral oncogene homolog	*FOS*	9.6 × 10^−5^	2.8
105	Frizzled homolog 6 (Drosophila)	*FZD6*	9.4 × 10^−11^	2.7
157	Wingless-type MMTV integration site family, member 4	*WNT4*	3.2 × 10^−8^	2.2
179	Matrix metallopeptidase 2	*MMP2*	3.5 × 10^−6^	2.1
183	Dickkopf homolog 3 (*Xenopus laevis*)	*DKK3*	4.7 × 10^−10^	2.1
206	Low-density lipoprotein receptor-related protein 1B	*LRP1B*	3.5 × 10^−6^	2.0
209	CD44 molecule (Indian blood group)	*CD44*	6.6 × 10^−8^	2.0

* Wnt genes were identified using the list provided at http://www.stanford.edu/group/nusselab/cgi-bin/wnt/ or http://www.rndsystems.com/molecule_group.aspx?g = 484&r = 2 or due to the attachment to the Gene Ontology term GO:0016055.

The expression of *LGR5* in ZG was confirmed by qPCR as 306-fold up-regulated compared with ZF (n = 18, *P* = 3.01 × 10^−9^). By contrast, in 12 APAs, qPCR found the expression of *LGR5* to be only 1.7% of that in the adjacent ZG. mRNA results were confirmed at the protein level through IHC using three commercial antisera raised against differing epitopes (Supplemental Figure 2A). IHC of key Wnt genes (β-catenin, the central signaling molecule of the canonical Wnt pathway; LEF1, the nuclear transcription factor activated by β-catenin; c-Jun, the target of the polarity noncanonical Wnt pathways; and phospho-c-Jun) in serial human adrenal sections showed ZG-selective distribution with colocalization of LGR5 (Figure 1B and Supplemental Figure 2B). In addition to the ZG-selective staining, some adrenals showed radial streaking into ZF for LGR5 in serial sections, reminiscent of the pattern previously associated with centripetal migration of ZG cells (Supplemental Figure 2A) (25, 26). Staining of the positive control, the small intestine, supported the specificity of LGR5 staining in the adrenals (Supplemental Figure 2C). Most β-catenin staining was membranous or cytoplasmic, with a few cells showing the nuclear staining induced by canonical Wnt activation (Figure 1B). Microarray analysis detected neither of the canonical Wnt ligands, Wnt1 or Wnt3a, as up-regulated in ZG, and their absolute expression in both adrenal zones were low.

### Transfection and treatment of *LGR5* and R-spondin-3 show reduction of aldosterone secretion in H295R cells and primary adrenals

Functional studies, overexpression and silencing of *LGR5* and treatment with its cognate ligand R-spondin-3, were performed on either the immortalized human adrenocortical H295R cell line or normal primary adrenal cells cultured from adrenalectomized adrenals of patients diagnosed with either primary hyperaldosteronism or pheochromocytoma.

**Figure 2.**
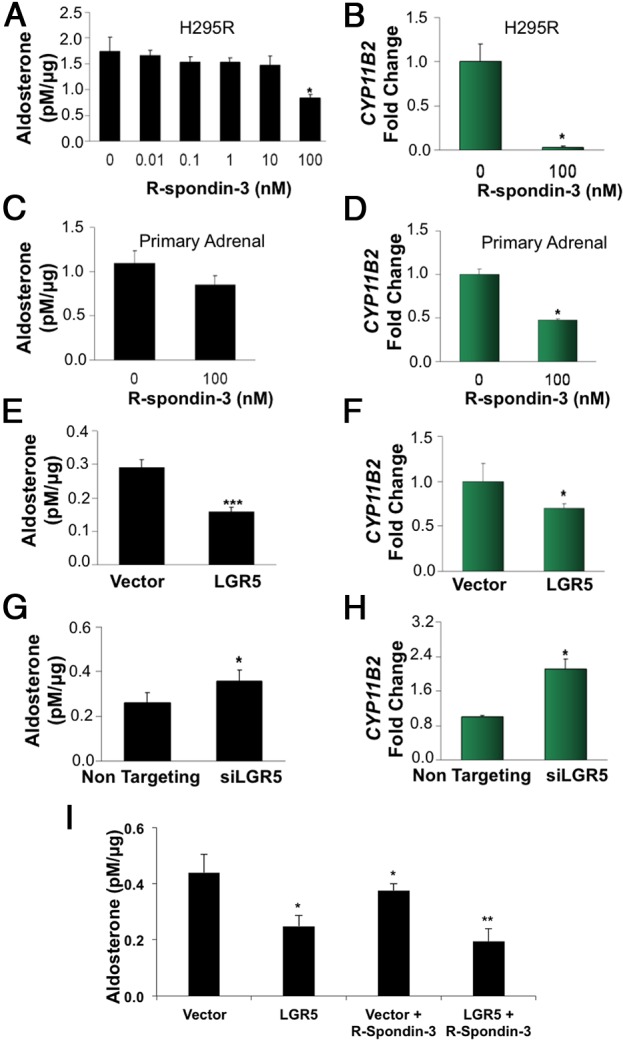
*LGR5* and its cognate ligand R-spondin-3 inhibit aldosterone production R-spondin-3 inhibition of aldosterone secretion (n = 12) (A) and *CYP11B2* transcription (n = 3) (B) by H295R cells and aldosterone secretion (n = 7) (C) and *CYP11B2* transcription (n = 3) (D) by normal human primary adrenal cells from patients with primary hyperaldosteronism. H295R and human primary adrenal cells were serum deprived in unsupplemented medium for 24 hours before exposed to vehicle control or R-spondin-3 as specified. Supernatants for aldosterone concentration measurement were collected after 8 hours incubation with drug, and cells were harvested for analysis of *CYP11B2* mRNA. *LGR5* transfection of H295R cells, showing inhibition of aldosterone secretion (n = 17) after 48 hours of transfection (E) and inhibition of *CYP11B2* transcription (n = 3) (F). pCMV6-AC-GFP was used as vector control (Vector). Silencing of *LGR5* in H295R cells (siLGR5) after 48 hours, showing stimulation of aldosterone (n = 7) (G) and increased CYP11B2 transcription (n = 3) (H). ON-TARGET plus non-targeting siRNA (nontargeting) transfected cells were used as experimental control. I, Additive effect of R-spondin-3 treatment on aldosterone production of *LGR5*-transfected H295R cells (n = 8). H295R cells were transfected with vector controls pCMV6-AC-GFP or GFP-tagged *LGR5* (Origene; 600 ng/μL). After 24 hours of transfection, H295R cells were serum deprived in unsupplemented medium for 24 hours, and further R-spondin-3 treatments were carried out with 8 hours of incubation from this point. Results are expressed in mean ± SEM. *P* values show significance between treatments and baseline control using a Student *t* test. *, *P* < .05; **, *P* < .01; ***, *P* < .001.

In both cell line and primary cultures, 100 nM of R-spondin-3 treatment caused a reduction in aldosterone secretion and *CYP11B2* expression (Figure 2, A–D). *LGR5* transfection similarly reduced aldosterone production (Figure 2, E and F). Conversely, silencing of *LGR5* caused an increase in aldosterone production and *CYP11B2* transcription (Figure 2, G and H). Some of the inhibition by R-spondin-3 and *LGR5* appeared due to reduction in cell number as seen on protein assay. However, even after protein correction, there was a reduction in aldosterone secretion, which was most marked for *LGR5*-transfected cells exposed to R-Spondin-3 (56% ± 5%, *P* = .002 Figure 2I).

### *LGR5* transfection in H295R cells shows reduction of cells

**Figure 3.**
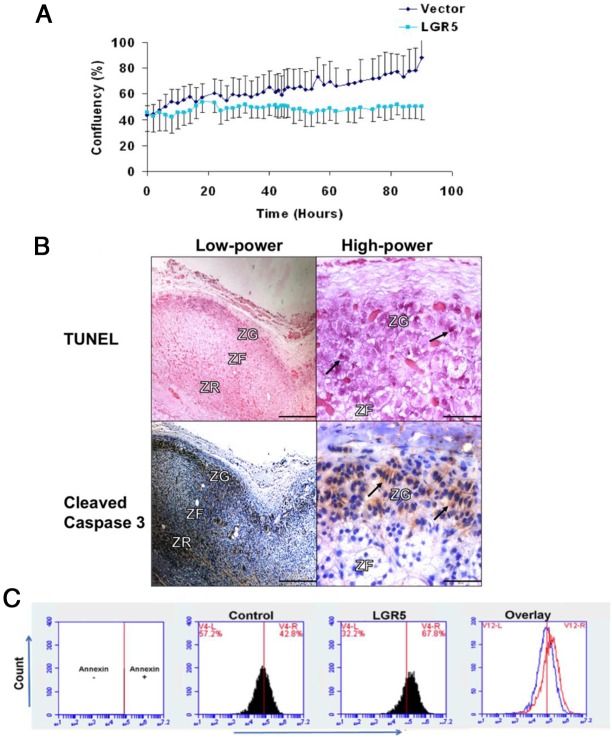
*LGR5* reduces human adrenocortical cell proliferation and increases apoptosis. A, Kinetic live-cell imaging compared *LGR5* and vector-transfected H295R cells (n = 6, *P* = 1.5 × 10^−5^ using a Student *t* test). B, Human adrenal section showing staining for TUNEL (apoptosis marker, dark purple) and cleaved caspase-3 (apoptosis marker, brown) in the ZG (as arrowed). Pictures are representative of six normal adrenal sections that were used in our microarray study: from four primary hyperaldosteronism patients and two pheochromocytoma patients. ZR, zona reticularis. Scale bar, 500 μm (low power) and 50 μm (high power). C, GFP-tagged, *LGR5*-transfected H295R cells undergoing apoptosis were annexin V stained for flow cytometry quantification. In the overlay, the blue line denotes the number of H295R cells stained with annexin V that were transfected with vector control, whereas the red line denotes the same for *LGR5*-transfected cells (n = 3, *P* = .009 using a Student *t* test).

We consistently noted a qualitative reduction in cell number and adhesion after *LGR5* transfection or R-spondin-3 stimulation. Kinetic live-cell imaging of *LGR5*-transfected cells directly quantified a 43% ± 11% reduction in the proliferation of H295R cells (*P* = 1.5 × 10^−5^) (Figure 3A), seen also in a cell viability assay (Supplemental Figure 3). These observations led us to investigate indices of proliferation and apoptosis in sections of human adrenal cortex and in *LGR5*-transfected H295R cells. In other species, the accepted model of adrenocortical cell fate has been of a unitary progenitor cell in the capsule that migrates centripetally through ZG and ZF to zona reticularis, in which it undergoes apoptosis (27, 28). In the absence of aldosterone production, eg, in the *CYP11B2*^−/−^ mouse, the processes of migration and apoptosis are accelerated (29). By contrast, in human adrenal, we found that the main site of apoptosis is ZG, as seen on both TUNEL and cleaved caspase-3 staining (Figure 3B and Supplemental Figure 4). Staining of H295R cells with annexin V and separation by flow cytometry quantified higher apoptosis in *LGR5*-transfected cells compared with vector controls (54% ± 3% vs 34% ± 3%; Figure 3C).

### *LGR5* activates AP1/Jun noncanonical Wnt pathway

**Figure 4.**
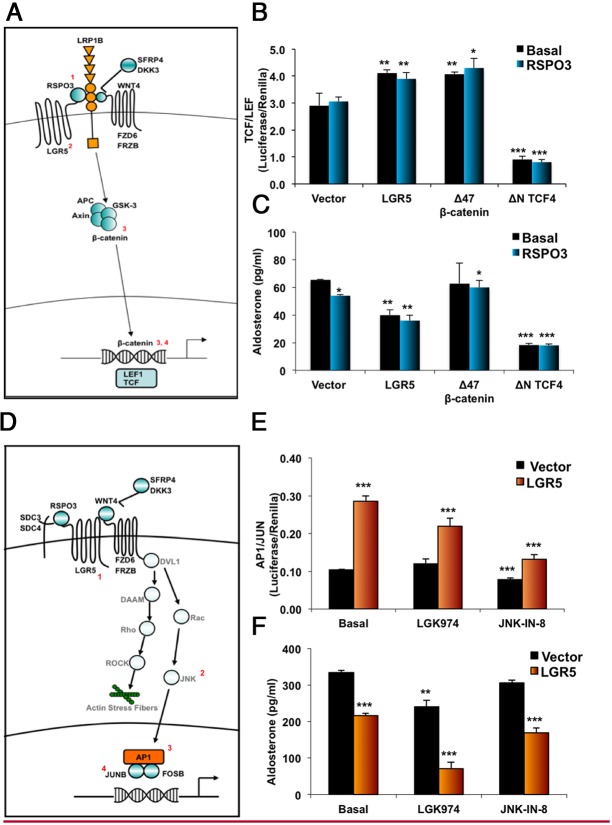
Dominant action of *LGR5* on the noncanonical (AP1/Jun) pathway. A, Schema showing genes expressed in the human adrenal from the canonical Wnt signaling pathway. Numbers (in red) show site of action of Wnt-related proteins, which were either used or measured in the experiment (1: R-spondin-3; 2: LGR5; 3: β-catenin; 4: TCF). B, Canonical Wnt signaling activity of H295R cells transfected with control vectors (Vector), *LGR5*, δN47 β-catenin (encoding a constitutively active β-catenin) or δN TCF4 (encoding a dominant negative TCF) were measured through cotransfection with a luciferase construct that had a TCF/LEF-responsive promoter (Cignal TCF/LEF reporter (luc) kit; SABiosciences). C, Aldosterone secretion during the same experiments (n = 5). D, Schema showing Wnt genes expressed in the adrenal from the noncanonical Wnt signaling pathway. Numbers (in red) show the site of action of Wnt-related proteins, which were either used or measured in the experiment (1: LGR5; 2: JNK; 3: AP1; 4: JUN). E, AP1/Jun noncanonical Wnt activity measured in the presence of LGK974 (porcupine inhibitor) and JNK-IN-8 (JNK inhibitor) (both 1 μM). F, Aldosterone secretion during the same experiment (n = 4). Results are expressed in mean ± SEM, and *P* values show significance between treatments and baseline control. *, *P* < .05; **, *P* < .01; ***, *P* < .001 using a Student *t* test.

Previous studies have shown that canonical Wnt activation increases proliferation and aldosterone production (15, 30). We hypothesized that the inhibition of aldosterone and net cell loss is due to *LGR5* inhibition of the canonical Wnt activation (31), although there have been previously postulated noncanonical Wnt effects in human adrenal (32). To determine which is the more likely mechanism, we cotransfected H295R cells with *LGR5* or a luciferase reporter with a promoter recognition site for either TCF/LEF, the canonical Wnt transcription factors, or one of the noncanonical Wnt targets, AP1/Jun or NFAT (Figure 4 and Supplemental Figure 5). In addition, we also investigated the effect on aldosterone production of a constitutively active or dominant-negative canonical Wnt pathway through transfection of a constitutively active β-catenin (δN47 β-catenin) or dominant-negative TCF (δN TCF4); and using small molecule inhibitors, we interrogated nonselective inhibition of Wnt signaling pathways (through inhibition of the Wnt chaperone porcupine) and selective inhibition of the noncanonical Wnt signaling pathway (through inhibition of JNK).

H295R cells, whose S45P mutant β-catenin is constitutively active, had the expected high basal levels of TCF/LEF activity, with only slight further activation on the addition of the δN47 β-catenin construct (Figure 4B). On the other hand, the δN TCF4 construct markedly reduced both TCF/LEF activity and aldosterone production (Figure 4, B and C). In contrast, transfection of *LGR5* caused a slight increase in TCF/LEF activity while decreasing aldosterone secretion (Figure 4, B and C). Supporting a predominant action of *LGR5* on a noncanonical Wnt pathway (Figure 4D), *LGR5* transfection increased AP1/Jun activity by almost 3-fold, and the noncanonical selective JNK inhibitor, JNK-IN-8, ameliorated this increase (Figure 4E). JNK-IN-8 inhibition, however, was insufficient to reverse *LGR5*'s mediated suppression of aldosterone secretion (Figure 4F), suggesting that AP1/Jun is not the only noncanonical pathway involved. The consequences of blocking Wnt signaling with the nonselective porcupine inhibitor, LGK974, had a similar inhibitory effect on aldosterone production as was seen with the TCF δN construct (Figure 4, E and F). *LGR5* had no significant influence on the NFAT reporter construct (Supplemental Figure 7).

## Discussion

We found the most-up-regulated gene in normal human ZG, compared with its adjacent ZF, to be *LGR5* and that its transfection into human adrenocortical cells results in the inhibition of aldosterone production, reduction in cell number, and activation of a noncanonical Wnt signaling pathway postulated as the mediator of the *LGR5* response.

Wnt signaling has well-documented roles in normal adrenal development, adrenocortical tumors, and H295R cells (30, 32, 33). However, no definite physiological role has been ascribed to Wnt in the regulation of aldosterone, especially in humans (34). The dominant inhibitory role of Wnt that we have found in human ZG helps to explain the lack of similarity between our microarray findings from the current study and those reported from adrenal tissues in which aldosterone production is stimulated. For example, none of the genes associated with the Wnt signaling pathway that were up-regulated in normal human ZG were up-regulated in our previous microarray analyses of APAs, one comparing APAs with adjacent adrenal, and the other comparing ZG-like APAs with more ZF-like APAs (6, 7). Furthermore, *LGR5* was almost absent from adjacent APAs in the present study. Finally, only one of our 18 Wnt related genes (*GPC3*) was similarly up-regulated in a microarray comparison of CYP11B2-positive cells in rat ZG with adjacent ZF (35). We believe that the key difference between human ZG on the one hand, and either adjacent APAs or rat ZG on the other, is that the dominant signaling pathway activated in the latter are those that stimulate aldosterone production, whereas the reverse appears true of human ZG.

We found that, unlike its reported effects in other tissues (23, 24), LGR5 in human adrenal cells stimulates predominantly noncanonical pathways. The AP1/Jun pathway, which was markedly stimulated by LGR5 transfection of H295R cells, influences cell migration (36). Its activation is also a measure for Rho/Rho-associated coiled-coil kinase regulation of both migration and polar cell polarity (see Figure 3D). The latter has not been studied in human ZG cells but could be key to the formation of glomerular structure and tight junctions because known genes associated with these structures (*TJP1* and *CLDN1*) were among our ZG-selective genes (Supplemental Figure 1). Our models of migration or adhesion did not detect an effect of *LGR5* (Supplemental Figure 6) but probably do not exclude their occurrence in the intact human adrenal. Intact adrenocortical structure was recently shown to be essential for the generation of the voltage oscillations that regulate aldosterone secretion (37, 38). We have not yet established which noncanonical Wnt pathway may be responsible for the inhibition by *LGR5* of aldosterone production because the AP1/Jun inhibitor, JNK-IN-8, had little impact on the inhibition.

Our study of ZG genes was prompted by the frequency of somatic mutations leading to common, small APAs. If aldosterone is required for ZG cell survival, as implied by *CYP11B2*^−/−^ mice (29), cells that mutate to protect aldosterone production will have a selective advantage. Most cells in human ZG do not express *CYP11B2* (39). The sparseness of *CYP11B2* in ZG is a general phenomenon whether adjacent to an APA or to a pheochromocytoma (39, 40). The most likely explanation of this down-regulation of *CYP11B2* is a physiological response to high salt intake. If physiological inhibition of aldosterone production has the same consequences as genetic deletion, as is suggested by our apoptosis data (Figure 3), then constitutive activation of aldosterone production after somatic mutations would confer a local selection advantage to mutated cells. Moreover, because the fate of ZG cells on centripetal migration is to proliferate and then disappear (26), the survival advantage to ZG cells may derive from entering a synthetic rather than a proliferative mode, providing an explanation as to why ZG-like APAs are often small.

Few completely normal adrenals are removed at surgery. We therefore restricted our choice to those adjacent to either an APA or pheochromocytoma, the most common indications for adrenalectomy in our hospital. These tumors have opposite effects on sodium balance, and future analyses will consider differences in expression between the adrenal adjacent to the two tumor types. Whereas, however, a limitation of our study design is the use of adrenals adjacent to a tumor, which may differ from completely normal adrenals, there was no difference in the expression of *LGR5* between these two groups of patients. It seems unlikely therefore that its abundance is due to the tumors. The immortalized adrenal cell line, H295R, is also not a perfect model for native ZG cells (41). Although primary adrenal cells are less easy to transfect in culture than H295R cells, we have supported our findings in the latter where possible using primary adrenal cells. The assumption taken is that although the ZG cells are a minority of the mixture of ZG, ZF, and zona reticularis cells, only the ZG cells contribute to the measurements of aldosterone production.

Our discovery suggests a hypothetical translational consequence, arising from evidence that the survival of ZG cells is dependent on the production of aldosterone (29). Current drug treatments for hyperaldosteronism antagonize aldosterone response but increase plasma aldosterone levels (3). Selective inhibition of aldosterone production has been problematic because of the 95% homology of *CYP11B1* (cortisol synthase enzyme) with *CYP11B2*. But the discovery of adrenally expressed genes whose mutation activates aldosterone production, and of adrenal pathways linked to reduced aldosterone secretion, provides novel targets, with potential for clinical development.

Chronic human diseases are often attributed to maladaptation when scarcity is replaced by plenty. We speculate that inhibition of aldosterone production by a R-spondin-3-*LGR5*-noncanonical Wnt signaling pathway creates a mechanism that is an appropriate response to excess salt but could paradoxically create a survival advantage for cells with somatic mutations causing autonomous aldosterone production. Identification of this pathway may now provide novel targets for the prevention and treatment of hyperaldosteronism.
